# Morinda citrifolia l. treatments (noni) reduce glycemia in the model of aloxan-induced diabetes in rats

**DOI:** 10.1186/1758-5996-7-S1-A45

**Published:** 2015-11-11

**Authors:** José Bégue Moreira de Carvalho, Glauce Socorro Barros Viana, José Galberto Martins da Costa, Thyciara Fontenele Marques, Alisson Cordeiro Moreira, Úrsula Tavares de Sousa, João Victor Rodrigues Lacerda, Enaide Soares Santos, Ítalo Cordeiro Moreira, Maria Janice Pereira Lopes, Maria Neyrilane Torquato de Souza

**Affiliations:** 1 Faculdade de Medicina Estácio de Juazeiro do Norte, Juazeiro do Norte, Brazil

## Background

Diabetes mellitus is a chronic disease characterized by hyperglycemia which Results from alterations in secretion or insulin action. There is a large number of studies searching for natural products potentially hypoglycemic and presenting a low risk of adverse effects.

## Objectives

To evaluate the effects of the hydro-alcoholic extract of fruits from M. citrifolia on the glycemic profile in rats.

## Materials and methods

Alloxan (40 mg/kg, iv) was injected in male Wistar rats (250 g) and after 48 h, animals were subjected to blood collection for measurements of blood glucose (mg/dL). Only those showing glycemia levels higher than 250 mg/dL were submitted to the study. The animals were daily administered with 100 and 500 mg/kg of the Noni extract (N100 e N500), 120 mg/kg Metformin (M120) and 120 mg/kg Metformin + 100 mg/kg Noni extract (M120+N100), orally for 1 month. After this period, animals were subjected to another blood collection for blood glucose determination. The untreated diabetic controls (DC) received saline for the same period. For statistical analysis, ANOVA and the Newmam-Keuls (post hoc test) were used for multiple comparisons. Whenever needed the Student “t” test was used for means comparison before and after treatments. The differences were considered significant at p<0.05.

## Results

The N100 and N500 groups reduced blood glucose by 63 and 70%, respectively, as related to glycemia levels before treatments (N100: n=7, before: 406,4±31,16; post-treatment: 149.7±47.57 and N500: n=9, before: 462.4±24.82; post-treatment: 136.7±22.6), while the M120, M120+N100 and DC groups showed decreases of blood glucose of only 28, 19 and 17%, respectively (M120: n=12, before: 319.8±12.27; post-treatment: 231.1±31.4; M120+N100: n=6, before: 343.0±20.21; post-treatment: 277,4±32,13 and DC: n=9, before: 411,4±15,79; post-treatment: 341.9±28.4).

## Conclusions

The repeated administration of the hydro-alcoholic extract from the fruits of Morinda citrifolia promoted a significant reduction in blood glucose, after 1 month at the doses 100 and 500 mg/kg, a result not observed after metformin, used as reference. Furthermore, no synergism was presented by the association of Noni extract with metformin, suggesting that Noni exerts its effects by a different mechanism of action. The chemical study of the extract revealed the presence, among others, of phenols in great concentrations and those may be, at least partly, responsible for the observed effects.

